# Plasma cytokine levels and HIV-specific immune responses during acute/early HIV infection

**DOI:** 10.1186/1742-4690-9-S2-P265

**Published:** 2012-09-13

**Authors:** G Turk, N Laufer, AM Rodriguez, Y Ghiglione, J Favilene, MJ Ruiz, O Sued, L Giavedoni, P Cahn, H Salomon, MM Gherardi

**Affiliations:** 1 Instituto de Investigaciones Biomédicas en Retrovirus y SIDA INBIRS, Buenos Aires, Argentina; 2 Instituto de Investigaciones Biomédicas en Retrovirus y SIDA INBIRS, Argentina; 3 Fundacion Huesped, Buenos Aires, Argentina; 4 Southwest National Primate Research Center, San Antonio, TX, USA; 5 Fundacion Huesped/Hospital Juan A Fernandez, Buenos Aires, Argentina

## Background

It is believed that initial encounter between HIV and the human host triggers a complex series of events that dictate future disease course. Inter-individual differences among the host-players involved in these processes seem to early determine different rates of disease progression. Here we were aimed at studying the relationship between innate and adaptive soluble immune mediators, HIV-specific T-cell response and the course of acute infection.

## Methods

Plasma levels of 37 cytokines were measured by Luminex technology in different groups of volunteers: 10 healthy donors (HD) and 50 HIV infected-subjects: 10 chronics, 12 aviremic controllers (EC) and 28 subjects enrolled during acute infection (AI). All HIV patients were off-HAART. Frozen PBMCs from the same individuals were used to determine HIV-specific T-cell responses by IFN-gamma ELISPOT. Data was compared inter- and intra-groups and correlated to viral load (VL), CD4 T cell counts and both virological (VL) and immunological (CD4 count) set-points (in AI), using parametric and non-parametric statistics.

## Results

Compared to HD, cytokines significantly elevated during acute and chronic infection included IL-1alfa, IL-10, IP-10 and TNF-alfa. Conversely, IL-12p40 and the macrophage-derived chemokine (MDC) were only significantly elevated in chronics and not in AI subjects who showed similar levels to HD and even EC. Moreover, levels of IL-12p40, IL-12p70 and MDC directly correlated with CD4 T-cell count among chronics and both CD4 T-cell count and immunological set point in AI. Regarding HIV-specific T-cell response during AI, proportion of Gag-specific and Nef-specific cells significantly correlated (directly and inversely, respectively) with immunological set point.

## Conclusion

Both early and late components of the immune system help preserve CD4 T-cell subset in HIV+ subjects: key cytokines involved in the initiation and regulation of cellular immune response and anti-Gag specificity of effector T-cells. These features should be taken into account during vaccine formulation design to boost favorable results.

